# A systems approach for analysis of high content screening assay data with topic modeling

**DOI:** 10.1186/1471-2105-14-S14-S11

**Published:** 2013-10-09

**Authors:** Halil Bisgin, Minjun Chen, Yuping Wang, Reagan Kelly, Hong Fang, Xiaowei Xu, Weida Tong

**Affiliations:** 1 Division of Bioinformatics and Biostatistics, National Center for Toxicological Research, US Food and Drug Administration, 3900 NCTR Road, Jefferson, AR 72079, USA; 2 Office of Scientific Coordination, National Center for Toxicological Research, US Food and Drug Administration, 3900 NCTR Road, Jefferson, AR 72079, USA; 3 Department of Information Science, University of Arkansas at Little Rock, 2801 S. University Ave., Little Rock, AR 72204-1099, USA

## Abstract

**Background:**

High Content Screening (HCS) has become an important tool for toxicity assessment, partly due to its advantage of handling multiple measurements simultaneously. This approach has provided insight and contributed to the understanding of systems biology at cellular level. To fully realize this potential, the simultaneously measured multiple endpoints from a live cell should be considered in a probabilistic relationship to assess the cell's condition to response stress from a treatment, which poses a great challenge to extract hidden knowledge and relationships from these measurements.

**Method:**

In this work, we applied a text mining method of Latent Dirichlet Allocation (LDA) to analyze cellular endpoints from *in vitro *HCS assays and related to the findings to *in vivo *histopathological observations. We measured multiple HCS assay endpoints for 122 drugs. Since LDA requires the data to be represented in document-term format, we first converted the continuous value of the measurements to the word frequency that can processed by the text mining tool. For each of the drugs, we generated a document for each of the 4 time points. Thus, we ended with 488 documents (drug-hour) each having different values for the 10 endpoints which are treated as words. We extracted three topics using LDA and examined these to identify diagnostic topics for 45 common drugs located *in vivo *experiments from the Japanese Toxicogenomics Project (TGP) observing their necrosis findings at 6 and 24 hours after treatment.

**Results:**

We found that assay endpoints assigned to particular topics were in concordance with the histopathology observed. Drugs showing necrosis at 6 hour were linked to severe damage events such as Steatosis, DNA Fragmentation, Mitochondrial Potential, and Lysosome Mass. DNA Damage and Apoptosis were associated with drugs causing necrosis at 24 hours, suggesting an interplay of the two pathways in these drugs. Drugs with no sign of necrosis we related to the Cell Loss and Nuclear Size assays, which is suggestive of hepatocyte regeneration.

**Conclusions:**

The evidence from this study suggests that topic modeling with LDA can enable us to interpret relationships of endpoints of *in vitro *assays along with an *in vivo *histological finding, necrosis. Effectiveness of this approach may add substantially to our understanding of systems biology.

## Background

Toxicity screening is an essential step in drug development since safety concerns have been one of the main causes of bottlenecks before drug approval [1,2]. *In vitro *assays, such as High Content Screening (HCS) methods, have become an important tool for safety screening. HCS has been actively evaluated for use in drug discovery due to the advantages of being high-throughput and requiring less physical material for testing.

Unlike conventional cytotoxicity assays, HCS offers the promise of understanding the biological functions underlying toxicity by simultaneously testing various cellular activities in live cells [3]. Providing temporal and spatial measurements of relations within the cell, HCS has gained acceptance in the research community and it has been actively applied over the past decade for the assessment of drug toxicity and study of mechanisms [4-11]. This so-called systems cell biology has also generated positive effects in the drug discovery process [12].

HCS has notable advantages over traditional cytotoxicity assays because it measures multiple cellular endpoints simultaneously so that it captures a more complete and dynamic picture of cellular response to an insult. We hypothesized that these endpoints together indicate the cell's condition under stress responding to a treatment in a probabilistic relationship. Such a characteristic can not be accurately described by most of the common approaches such as clustering or PCA and should be modeled with a Bayesian relationship. Unfortunately, most, if not all, post-experiment analysis often involves building discriminative models that use each read-out assay (i.e., endpoint) as an independent feature. Specifically, these data analyses treat individual endpoints as independent features rather than observing their interdependencies in a probabilistic relationship [13]. For example, O'Brien *et al. *reported an HCS assay based on the HepG2 cell line and paired the HCS endpoints with the conventional *in vitro *cytotoxicity assay in a one-to-one comparison to assess the human hepatotoxicity of the tested drugs [3]. Likewise, Xu *et al. *generated eight cellular measurements in an HCS assay based on rat primary hepatocytes and employed a Boolean logical OR to indentify individual endpoints with high predictivity for clinical drug-induced liver injury [14]. As promising as these results are, this practice does not take full advantage of interdependencies among these cellular endpoints indicated by the systems biology of the cell.

In order to best use the multi-parameter measurements of live cells that HCS assays provide, a statistical analysis method must have the capability to extract hidden knowledge and relationships from these measurements. The best way to address this issue is to adapt a systems approach that would not only model relations between endpoints, but also link such a relationship to elucidate the cellular events leading to toxicity [15]. For this reason, we investigated a statistical model which attempts to both summarize cellular events reflected in the endpoints measured in a parallel fashion in HCS and establish a global understanding of their relations in the cell.

We used Latent Dirichlet Allocation (LDA) [16] for topic modeling, which has primarily been applied to problems in text mining [17-22], to analyze the data from the HCS assays. LDA assumes that the expression of the HCS endpoints follow a probabilistic distribution and can be modeled by the mathematic expression of "topics" that consist of these endpoints. The topic model allows endpoints to be linked to multiple topics with different strength levels. Similarly, it builds probabilistic associations between topics and drugs, which we treat as documents containing occurrences of endpoint measurements (i.e., words). Thus, LDA acts as more than a classification or clustering approach and instead aids in the interpretation of the topics.

In this work, we built a topic model using LDA for rat primary hepatocyte-based HCS assays to investigate the relationship of the cellular level response to the drug treatment observed in this assay and the liver injury related necrosis observed in the whole animal (*in vivo*) study. Our study demonstrated the utility of topic modeling, including the innate properties of the assay, to interpret the HCS results and thus reach a better understanding of the toxic response. The results indicate that endpoints under significant topics corresponded to the cellular mechanisms involved in the progression of hepatocellular necrosis *in vivo *as well as recovery from liver injury. This proof-of-concept study demonstrates that topic modeling has the potential to model biological data beyond simply text documents to exploit the relationships of assay endpoints.

## Materials and methods

### HCS assays

A set of compounds with a wide range of known mechanisms of action was chosen to test the range of detection of the mechanistic profiling assays applying the cellular systems biology (CSB™) approach (CellCiphr^® ^profile). Eight endpoints, Cell loss, Nuclear Size, DNA Damage, Apoptosis, Lysosomal Mass, DNA Fragmentation, Mitochondrial Potential, and Steatosis were measured simultaneously in populations of cultured rat primary hepatocytes at multiple time points to profile both the potency and specificity of the cellular toxicological responses [23]. Briefly, rat primary hepatocytes were prepared using the method reported by Berry *et al. *[24]. Cell viability obtained from this method ranged from 85% to 95%. Diluted test compound solutions were added to each well at identical final concentrations. The maximal concentration of treatment was 200 μM with 10-point titrations for each compound using a 2-fold dilution series and tested up to 48 hours. The final concentration of DMSO in each well was 1% (v/v). For all assays, cells were analyzed using an ArrayScan VTI HCS Reader in the high-resolution mode with a 10×/0.45 NA objective and a 0.63 × coupler.

### *In Vivo *data from animal experimental study

The necrosis data used in this study was obtained from the Japanese Toxicogenomics Project (TGP). Details regarding the animal study protocol are available elsewhere [25,26]. Briefly, male Sprague-Dawley rats were purchased from Charles River Japan, Inc. (Kanagawa, Japan). The TGP selected a set of compounds to test. Each group of animals was administered at low, middle and high doses with the concurrent control group. The maximum tolerated dose (MTD) of each compound was determined by one week dose range finding (DRF) study and set as the high dose. Low and middle doses were 1/10 and 1/3 of high dose, respectively. Animals were administered a single dose and then sacrificed at 3, 6, 9, and 24 hours after dosing. Liver samples were immediately collected from the left lateral lobe of the livers and processed through dehydration and embedded in paraffin block for slide preparation and observation of histopathology. Histopathological changes were examined at four well recognized institutions in Japan by certified pathologists. Alterations of histology were described using the standard terminology unified by "the Japan Toxicology Society of Pathology" which can be found at (http://www.nihs.go.jp/center/yougo/15.pdf).

### Data preprocessing

**Figure 1:**
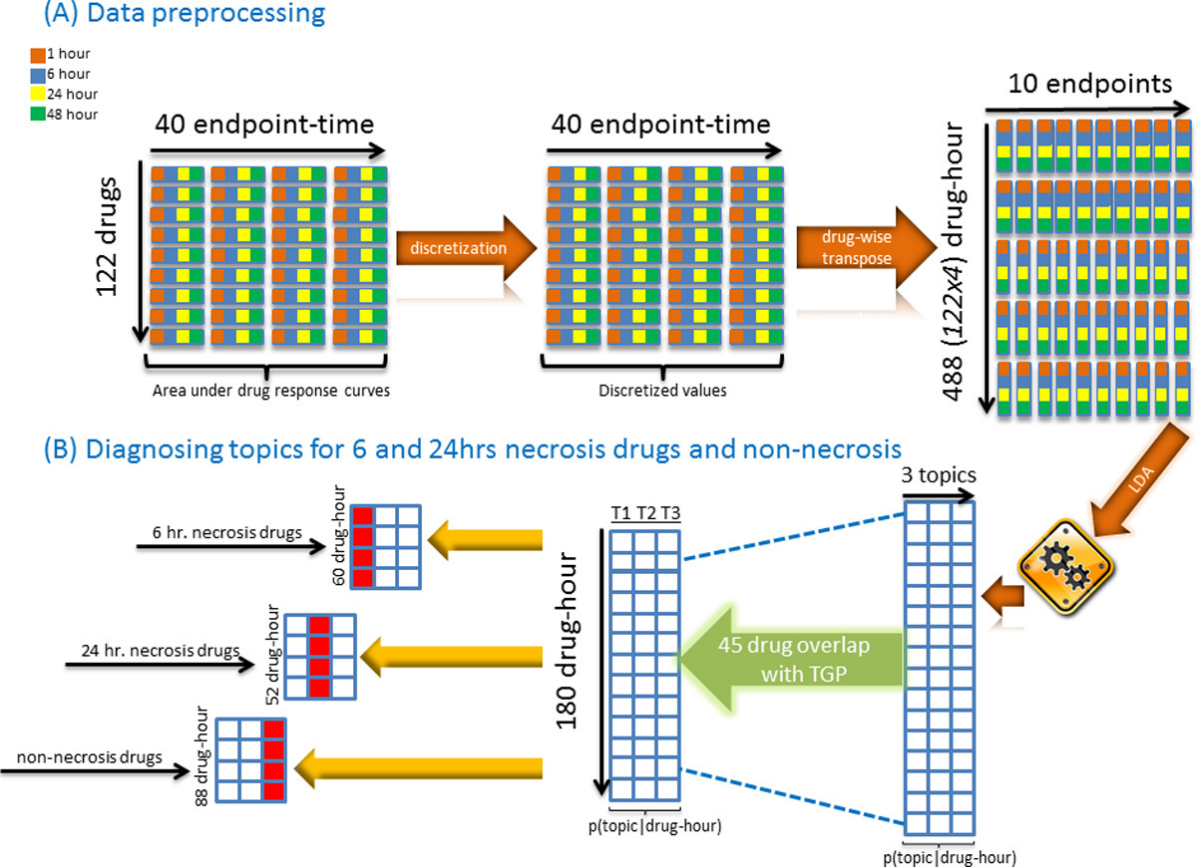
**Flow chart of the procedure of this study, from data processing to topic diagnosis**. (A) steps include discretization and change in the orientation of the input matrix for LDA. (B) steps developed parameters for different class definitions in Settings I, II, and III (Table 1), and identified diagnostic topics for the 45 drugs in the *in vivo *histopathology dataset.

The HCS assays included 8 endpoints (Steatosis, DNA Fragmentation, Mitochondrial Potential, Lysosome Mass, DNA Damage, Apoptosis, Cell Loss, and Nuclear Size) that were observed for 1, 6, 24, and 48 hours after treatment of 122 drugs (Additional file 1). These endpoints were measured in two plates with DNA Damage, Apoptosis, Lysosome Mass, and DNA Fragmentation at the first plate and Mitochondrial Potential and Steatosis at the second plate, while Cell Loss and Nuclear Size were measured at both plates. In order to incorporate all the assay measurements into our model, we treated Cell Loss and Nuclear Size in the second plate as two different endpoints. This resulted in 10 endpoints in total. Different dose levels were imposed which constituted a dose-response curve for each endpoint at the time of measure. We normalized each dose-response curve by its corresponding curve obtained through Dimethyl Sulfoxide (DMSO) solvent (control). The normalization step was followed by quantifying these cellular responses where area under the dose-response curve (AUC) was calculated by using a numerical integration approach. Subsequently, for each drug we produced a data table where every column represented a time point of an endpoint (left matrix in Figure 1A). Including the replicated plates for Cell Loss and Nuclear Size, 40 AUC values were obtained for each drug.

Since the topic modeling approach assumes all the entries of data are generated by a multinomial distribution, continuous AUC values were converted into integers (middle matrix in Figure 1A) by a discretization method [27] that uses a binning approach. Entries from each column were divided into 100 bins and mapped to the corresponding integer. In this new numerical representation, each cell provided approximate information on the frequency of the corresponding column variable.

In order to observe the endpoint behavior over time, we changed the orientation of the data so that rows became the drug-hour combinations. Therefore, number of columns shrank to number of endpoints which was 10 including the replicates. This perspective provided a temporal observation which could be exploited by topic modeling. In other words, each drug-hour stood for a document and values in the corresponding row quantified the number of occurrences of endpoints (words) in that document. The analogy we carried out here allowed us to use 10 endpoints as a vocabulary and construct different profiles (documents) for every time step of each compound (right matrix in Figure 1A).

### Topic modeling

In this study, we used Latent Dirichlet Allocation (LDA) as a topic modeling tool, which is an improved version of earlier models [28,29] and allows multiple topic assignments [16]. LDA assumes that every document is a mixture of different topics which govern the allocation of words across documents. The model formalizes this principle by posing a multinomial distribution of topics, *z*, over words, i.e., *z*~Mult(*θ*) with parameter *θ*. To make the likelihood estimations computationally tractable, *θ *is assumed to follow a Dirichlet distribution which is the conjugate prior of multinomial distribution. Therefore a hyperparameter, *α*, is introduced and *θ *is sampled from Dir(*α*). Similarly, word distributions are also controlled by another multinomial distribution for given topics and parameters having Dirichlet distribution which takes *β *as another hyperparameter. For a document which has *N *words, this generative process can be concisely presented as follows [16]:

1. Choose *θ ~ *Dir(*α*).

2. For each of the words *w_n _*where n∈{1,…,N}

a. Choose a topic z_*n *_~ Mult(*θ*)

b. Choose a word *w_n _*from another multinomial distribution that is conditioned on the topic z_*n *_and a *prior β*. i.e., p(*w_n_*| z_*n*, _*β*)

A distinguishing feature of LDA is that it can assign an unseen document to discovered topics. Furthermore, it provides interpretable conditional probability tables (CPT) for aforementioned associations such as document-topic (p(topic|document)) and word-topic (p(word|topic)). CPTs not only give the mixture weights of topics for given documents, but also tell how likely it is that a word comes from a given topic.

### Diagnostic topic

CPTs can be used to measure the document-topic associations. These probabilistic values can also be used to rank the most probable topics. However, there is a need to measure *diagnostic topics *[30] for a class of documents instead of an individual treatment. Griffiths and Steyvers introduced this term to link a class of documents to their statistically significant topic or diagnostic (indicator) topic [30]. The methodology first requires calculation of the mean probabilities of each category for a given topic *k*. Secondly, each class (*c*) mean for topic *k*, $\mu_{ck}$, is divided by the sum of all class means for topic *k*. If there are *C *categories, the following expression gives the significance score of topic *k*, for a class *c*.

(1) $$S_{ck}=\frac{\mu_{ck}}{\overset{C}{\underset{c=1}{\sum }}\mu_{ck}}$$

Finally, we have a *C*x*K *matrix in which rows are the scores for topics and we declare k* as the diagnostic topic for a group *c *if $k^{*}=\underset{k}{\text{arg}}\text{max}S_{ck}$.

## Results

### Study design

One of the goals in toxicogenomics is to link *in vitro *findings with *in vivo *study in order to better understand the mechanisms underlying toxic insult. Importantly, the concordance of the *in vivo *and *in vitro *results is a key indicator of potential to replace and reduce the animal uses in assessing risk of a broad range of medical and pharmaceutical products. Implementation of the model on HCS data was followed by analyzing 45 compounds whose histopathological data and HCA data available for this study, where the histopathological data was obtained from the TGP database while the HCS study was conducted by a commercial vendor (Additional file 2). We split drugs into groups based on whether they caused necrosis at 6 or 24 hour after treatment. This split resulted in three groups: 22 drugs with no necrosis finding at either time point, 15 drugs showing necrosis at 6 hours, and 13 drugs showing necrosis at 24 hours (Additional file 3). Corresponding settings in Table 1 were used to detect diagnostic topics, a concept which was originally developed for detecting topics that match with documents that share a commonality. Similarly, we extend this notion to identifying topics to observe their agreement with necrosis vs. non-necrosis findings at either 6 or 24 hours. Figure 1B illustrates how the settings below were incorporated with CPT (p(topic|drug-hour)) of 45 common drugs.

**Table 1:** Settings for diagnostic topics

	Necrosis (# of drugs)	Non-Necrosis (# of drugs)
**Setting I**	Necrosis finding at 6^th ^hour (15)	No necrosis finding at 6^th ^hour (30)
**Setting II**	Necrosis finding at 24^th ^hour (13)	No necrosis finding at 24^th ^hour (32)
**Setting III**	Necrosis at 6^th ^or 24^th ^hour (23)	No necrosis at either 6^th ^or 24^th ^hour (22)

In order to see whether the model can distinguish three groups (6hr necrosis, 24hr necrosis, and non-necrosis), we set the number of topics equal to three for the vocabulary of 10 endpoints (terms). Next, using the implementation by Blei *et al. *[31], we developed the model for 488 drug-hour combinations (four time points each for the 122 compounds). The resulting conditional probability tables (CPTs) were used in three analyses: i) grouping and ranking endpoints within topics, ii) diagnostic topic identification, and iii) linking endpoints to cellular processes.

### Groupings and ranking of endpoints

The topics (*t*) not only grouped the endpoints based on their distribution across drugs, but also provided an importance measure with the probability scores of endpoints (*e*) for given topics, *p(e|t)*. More specifically, *p(e|t) *indicates which endpoints are more important for that particular topic. In Table 2, the endpoints for each topic are listed with the endpoints ordered based on the strength of the relation. Notice that replicated endpoints are distinguished with indices in parentheses.

**Table 2:** Endpoint rankings for topics

Topic 1	P(*e*|*t*)	Topic 2	P(*e*|*t*)	Topic 3	P(*e*|*t*)
Steatosis	0.221	DNA Damage	0.251	Cell Loss (2)	0.191
DNA Fragmentation	0.192	Apoptosis	0.191	Cell Loss (1)	0.190
Mitochondrial Potential	0.175	DNA Fragmentation	0.129	Nuclear Size (2)	0.179
Lysosome Mass	0.122	Mitochondrial Potential	0.124	Nuclear Size (1)	0.171
Cell Loss (1)	0.109	Nuclear Size (2)	0.103	Mitochondrial Potential	0.144
Cell Loss (2)	0.094	Nuclear Size (1)	0.095	Lysosome Mass	0.086
Apoptosis	0.033	Lysosome Mass	0.029	DNA Fragmentation	0.020
DNA Damage	0.025	Cell Loss (1)	0.026	Steatosis	0.010
Nuclear Size (2)	0.021	Steatosis	0.026	Apoptosis	0.004
Nuclear Size (1)	0.009	Cell Loss (2)	0.025	DNA Damage	0.004

Each topic contains a ranked list of 10 endpoints, but these can be separated into disjoint groups by comparing *p(e|t) *values. Namely, we compared three probabilities *p*(*e*|Topic 1), *p*(*e*|Topic 2), and *p*(*e*|Topic 3) for each *e *and assigned it to the topic with the highest probability. In doing so, we identified groups of terms with the highest association to their topics and underlined them in Table 2. Steatosis, DNA Fragmentation, Mitochodrial Potential, and Lysosome Mass were highly significant for Topic 1. Similarly, DNA Damage and Apoptosis were the most highly associated endpoint terms for Topic 2. The remaining terms, Cell Loss and Nuclear Size fell under Topic 3.

### Diagnostic topics for necrosis vs. non-necrosis

The identification of diagnostic topics requires at least binary class labels so that the score $S_{ck}$ can be calculated. In this case, we used histopathological data for 45 drugs generated by the TGP database to establish the Settings I, II, and III corresponding to temporal observation of necrosis and non-necrosis drugs. In each setting, necrosis observations determined two classes, i.e., necrosis vs. non-necrosis. Plugging CPTs (p(topic|drug-hour)) generated by LDA into Eq. 1, which also uses the class label, we obtained scores for each topic. In Table 3, we summarize all settings with their diagnostic topics. For instance, Setting I consistently favors Topic 1 because its score is always the greatest among the three topics. This implies that Topic 1 is the diagnostic topic for drugs causing necrosis in rats at 6 hours. Similarly, Topic 2 appears to be the diagnostic topic for the drug group associated with necrosis at 24 hours in Setting II below. On the other hand, Setting III presents a clear cut design where the classes were defined for the drugs that were never involved in necrosis at 6 and 24 hours. For the 1^st ^hour in the non-necrosis group, Topic 1 gives the highest score. However, Topic 3 becomes the diagnostic topic for these drugs, representing an agreement between *in vivo *and *in vitro *data for non-necrosis behavior at 6 and 24 hours. Finally, Topic 3 can be claimed as the representative topic for non-necrosis drugs.

**Table 3:** Scores for diagnostic topics

*in vitro* *in vivo*	Time points (HCS Assay)	Topic 1	Topic 2	Topic 3
**6^th ^hr. necrosis** **(Setting I)**	1^st ^Hour	**0.553**	0.477	0.481
	
	6^th ^Hour	**0.564**	0.552	0.490
	
	24^th ^Hour	**0.612**	0.462	0.487
	
	48^th ^Hour	**0.571**	0.277	0.499

**24^th ^hr. necrosis** **(Setting II)**	1^st ^Hour	0.41	**0.58**	0.52
	
	6^th ^Hour	0.45	**0.57**	0.50
	
	24^th ^Hour	0.48	**0.66**	0.49
	
	48^th ^Hour	0.47	**0.89**	0.48

**Non-necrosis drugs for 6^th ^and 24^th ^hrs** **(Setting III)**	1^st ^Hour	**0.521**	0.450	0.502
	
	6^th ^Hour	0.459	0.404	**0.513**
	
	24^th ^Hour	0.398	0.371	**0.522**
	
	48^th ^Hour	0.439	0.187	**0.520**

### Topics as bridging components

**Figure 2:**
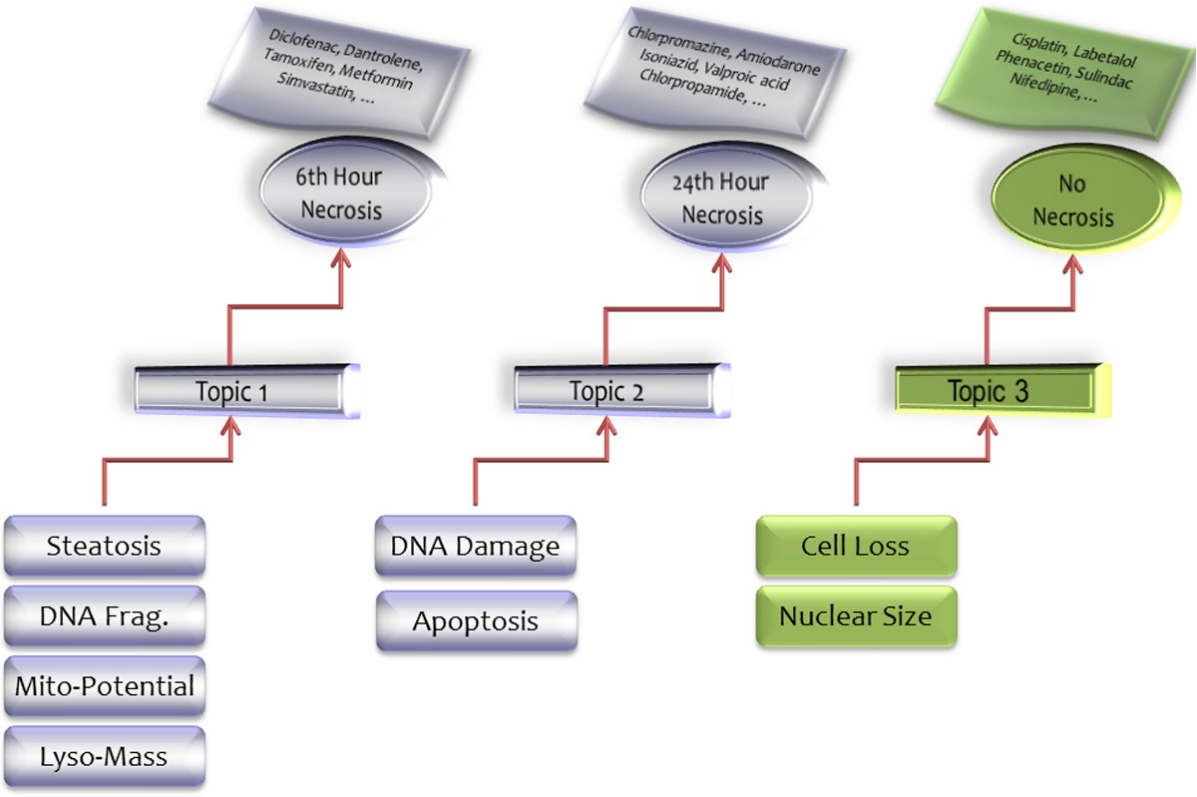
Transitive relations from in vitro assays and in vitro histopathological observations over topics

We assigned the endpoints to topics to form disjoint clusters and determined diagnostic topics to represent drug groups for different settings. In both analyses, we used topics as auxiliary variables. By means of topics, we further linked endpoint groups from HCS data to drug groups defined in the three necrosis conditions. We followed this process to demonstrate that topics could be also used to bridge *in vitro *HCS data and *in vivo *histopatholocal findings as illustrated in Figure 2. For instance, Steatosis, DNA Fragmentation, Mitochondrial Potential, and Lysosome Mass are listed under Topic 1, which corresponds to 6^th ^hour necrosis drugs from TGP. Drugs with necrosis appearing in the 24^th ^hour from the *in vivo *study are associated with Topic 2, which contains DNA Damage and Apoptosis. Lastly, Topic 3 both indicates non-necrosis and consists of the remaining endpoints, Cell Loss and Nuclear Size. These links, finally, enable us to build transitive relationships between endpoints from the *in vitro *HCS data and necrosis findings *in vivo*.

## Discussion

HCS offers impressive throughput because of its parallel read outs for multiple endpoints. This approach has recently been favored as a new technology in cell systems biology [4-11] and has been demonstrated in various applications. However, this approach can further benefit from an improved bioinformatics approach, considering the interdependencies of endpoints, which is an innate property of HCS. Although methods like HCA, PCA, k-means, and SOM are commonly used to identify natural groupings of samples, topic modeling offers different aspect of results, which use conditional probabilities to highlight importance of any component we studied (time points, assay types, and endpoints). Furthermore, its probabilistic nature allows samples to be assigned to multiple clusters, even though we used these conditional probabilities to obtain mutually-exclusive endpoint clusters. A limitation of this methodology is the assumption that the data values are governed by a multinomial distribution which may not be fully appropriate for continuous data. Since a continuous probability function in such a setting is not computationally tractable, biological data were often discretized in earlier studies [32,33]. Similarly, we have demonstrated the application of Latent Dirichlet Allocation (LDA) to HCS data.

As a proof-of-concept study, we introduced a methodology that is rooted in text mining, but by analogy could be efficiently carried out in the analysis of HCS data. Similar to the fact that a text is a mixture of topics; the measurement of endpoints for a given drug can be considered as a consequence of multiple cellular interactions. Thus, cellular responses to each compound at a particular time point were considered as a document for topic modeling. Once we applied LDA to the document-term representation for topic modeling, we obtained two probability measures in which topics played an intermediate role. That is to say, an ordered list of endpoints for given topics was generated along with topic probabilities of each drug.

In this study, topic-based probabilistic associations were interpreted in the context of necrosis findings observed by histopathological examinations from rats. In particular, necrosis was used as a criterion to determine the diagnostic topics, and drugs were categorized into those causing necrosis at 6 hours, causing necrosis at 24 hours, or not causing necrosis at either 6 or 24 hours. The purpose of this process was to match a group of cellular events caused by these groups of drugs to the progression of necrosis found in *in vivo *experiments. Results demonstrated a one-to-one correspondence between diagnostic topics and groups of drugs with similar necrosis profiles.

Acquisition of topics by LDA has the advantage of associating each term (endpoint) to multiple topics where they can be sorted based on probabilities. In other words, every topic consists of the same endpoints with different orders and endpoints are not forced to be assigned to a single cluster as it happens in *k*-means and hierarchical clustering methods. This is reasonable since none of the biological events occur independently rather in order by probabilistic significance. However, we split the endpoints into disjoint sets after showing their importance for given topics. For this reason, endpoints were assigned to their most probable topics regarding their rankings providing potentially important clues as to the cellular processes underlying a necrotic response to toxic agents. We illustrated how topics could link endpoints to a group of drugs in Figure 2 where different cellular events might be related to histopathological observations. Applying this methodology to the data here we observed the utility of the approach to interpreting HCS results.

Topic 1 was assigned as the diagnostic topic for necrosis at 6 hours, and was associated with the endpoints Steatosis, DNA Fragmentation, Mitochondrial Potential, and Lysosome Mass. Changes in DNA Fragmentation are characteristic of necrotic cell death, where the presence of 5' overhangs are seen, and changes in Lysosome Mass and Mitochondrial Potential are consistent with the changes to cell ion permeability that eventually lead to cell rupture [34,35]. Steatosis may either be evidence of the dysregulation of cellular transport or may itself be the cause of necrosis if large amounts of lipid distort the cell to the point of rupture [36].

Topic 2 was associated with DNA Damage and Apoptosis, which were diagnostic for necrosis at 24 hours. The longer time after exposure reveals the difference between more and less rapid-acting compounds. These results are somewhat perplexing given the differing pathological mechanisms underlying necrosis and apoptosis, although they do share common features such as membrane potential dysregulation [36,37]. However, it may be possible that the initial round of necrosis leads to a round of apoptosis in the remaining cells due to changes in the extracellular environment or that the length of exposure necessary to initiate the apoptotic response in those conditions is longer than the six hour time point.

Cell Loss and Nuclear Size showed a highly significant connection with Topic 3, which was an indicator of non-necrosis drugs as shown in Table 3. This could be considered as a biological confirmation of less-toxic events and indication of hepatocyte regeneration. Cell growth (increase in cell mass) and cell proliferation (increase in cell number) are usually coordinated to ensure that cell size is properly maintained. Hepatocytes are unique among differentiated parenchymal cells because they retain a stem cell-like ability to proliferate. This property remains in rat hepatocytes in primary culture and underlies the remarkable capacity of the liver to regenerate following acute injuries that diminish hepatic mass [38]. Hepatocyte regeneration proceeds along a sequence of distinctive phases and requires priming of hepatocytes to achieve competence for proliferation, such as increasing synthesis of RNA and proteins. Thus, hepatocytes increase in size at the early stage of the cell cycle, and the change of nuclear size is proportional to the change of cellular size [39]. Generally, the nucleus increases in size from the time of its formation [40]. In addition, it is reported that hypertrophy precedes proliferation in liver regeneration, suggesting that the first response to liver injury is an enlargement in hepatocyte size [41].

All endpoints measured in the HCS assay here are essential for toxicity assessments. In that sense, besides an independent analysis or a pair-wise comparison, it is important to interpret how these events lead to toxicity. For this reason, we not only used LDA to retrieve probabilistic associations of each endpoint to topics, but also incorporated a histopathological assessment of necrosis to test whether there is any biological meaning hidden behind these topics. For example, the drugs that caused necrosis in rats after 6 hours of treatment were also observed with significant changes of *in vitro *measurement in some pre-lethal endpoints including Steatosis, DNA Fragmentation, Mitochondrial Potential and Lysosome Mass, while the drugs that caused necrosis after 24 hours were associated with the cellular events in DNA Damage and Apoptosis. Obviously, the former observation in the cellular assay seems to reveal more acute injury, while the latter one reflects the cell death that might correlate the necrosis in rats observed even after 24 hour treatment. In other words, our incorporation of *in vivo *histopathology data over time with data from HCS agreed with the conventional wisdom regarding the cause of toxic necrosis.

Although they involve different practices and meanings, *in vitro *and *in vivo *data should be considered complements of each other. One of the goals here was to make use of these two data types to reveal biological facts. Besides using a novel computational tool to analyze HCS, we provide an example of a way to efficiently bridge *in vivo *and *in vitro *data by means of topic. By using this intermediate variable, we were able to correlate histopathological findings with the results from the HCS assays. The ability of the model to discover patterns with an unsupervised nature indicates its potential to be an alternative approach for analyzing HCS data. Hence, one direct application of this methodology for us is the early detection of drug-induced liver injury by interpreting the HCS content under probabilistic measures for drugs and endpoints. We intend to apply this method to predict the DILI potential of drugs by not only considering a single endpoint, but relying on the full set of data generated by HCS.

## Conclusion

We have presented here a systems approach that is capable of integration of multiple measurements from High Content Screening (HCS) by considering the interdependencies across endpoints. By analogy with text mining, endpoint distributions and proportions across topics were used to gain insight into the content of *in vitro *data. Further, discovered relations were analyzed along with corresponding *in vivo *data. The results showed that Latent Dirichlet Allocation (LDA) could improve the interpretation of HCS data for use in systems biology. The agreement we observed between *in vitro *and *in vivo *data through topics obtained by LDA provide early evidence for the effectiveness of this strategy.

## Disclaimer

The findings and conclusions in this article have not been formally disseminated by the US Food and Drug Administration (FDA) and should not be construed to represent the FDA determination or policy.

## Competing interests

The authors declare that they have no competing interests.

## Authors' contributions

HB performed all calculations and data analysis, and wrote the first draft of the manuscript. WT and XX had the original idea, developed the methods, and guided the data analysis and presentation of results. MC, YW, RK, and HF contributed to the data analysis, verified the calculations, and assisted with writing the manuscript. All authors read and approved the final manuscript.

## Supplementary Material

Additional file 1 Click here for file

Additional file 2 Click here for file

Additional file 3 Click here for file

## Declarations

Publication costs of this article were funded by the US government.

This article has been published as part of *BMC Bioinformatics *Volume 14 Supplement 14, 2013: Proceedings of the Tenth Annual MCBIOS Conference. Discovery in a sea of data. The full contents of the supplement are available online at http://www.biomedcentral.com/bmcbioinformatics/supplements/14/S14.
